# Pan-genome analysis of soybean terpene synthase identifies *GmTPS20* as a defense-related linalool synthase

**DOI:** 10.3389/fpls.2026.1845603

**Published:** 2026-05-15

**Authors:** Taotao Han, Haitao Liu, Tianhao Lu, Cheng Jiang, Chengrun Wang, Guopeng Miao

**Affiliations:** 1 Department of Bioengineering, Huainan Normal University, Huainan, China; 2 Key Laboratory of Bioresource and Environmental Biotechnology of Anhui Higher Education Institutes, Huainan Normal University, Huainan, China

**Keywords:** linalool synthase, metabolic engineering, pan-genome, soybean, terpene synthase

## Abstract

Soybean terpene synthase (TPS) genes are pivotal to ecological adaptation and stress resilience, yet their genetic diversity, evolutionary history, and enzymatic mechanisms remain poorly understood. A pan-genomic survey of 27 soybean genomes was performed to identify and classify TPS loci by conservation status. Phylogenetic reconstruction and Ka/Ks analysis were used to infer evolutionary relationships and selection regimes. An uncharacterized core gene, *GmTPS20*, and its close homolog *GmTPS15* were prioritized for functional characterization using expression profiling, subcellular localization, in vitro enzyme assays with geranyl diphosphate (GPP), neryl diphosphate (NPP), and farnesyl diphosphate (FPP) isomers, transient expression in *Nicotiana benthamiana*, and structural docking. The pan-genome survey identified 26 TPS loci: 15 core, four near-core, five variable, and two private, highlighting strong purifying selection on conserved members alongside lineage-specific losses. Soybean TPSs fell within the TPS-a, TPS-b, TPS-c, TPS-e/f, and TPS-g clades, with most loci exhibiting Ka/Ks < 1. *GmTPS20* showed broad expression peaking in young leaves and was induced by insect herbivory and methyl jasmonate, whereas *GmTPS15* was enriched in reproductive tissues. Both proteins localized to chloroplasts, consistent with the MEP pathway. *GmTPS20* acted as a substrate-specific monoterpene synthase that converted GPP to linalool and NPP to linalool and nerol, but did not accept FPP isomers; transient expression in *N. benthamiana* confirmed linalool accumulation in planta. Under matched conditions, *GmTPS15* produced no detectable volatile products. Structural docking indicated that both enzymes can bind GPP; however, *GmTPS20* features a more compact diphosphate-coordination network and a deeper, narrower active site, whereas *GmTPS15* adopts a more open pocket with reduced polar constraints, rationalizing their divergent catalytic behaviors. Collectively, these findings clarify mechanisms of TPS functional diversification in legumes and provide molecular targets for engineering terpenoid-based defense and desirable agronomic traits in soybean.

## 1 Introduction

Plant secondary metabolites play pivotal roles in mediating plant-environment interactions, functioning both as chemical defenses and as ecological signaling molecules (Dixon and Dickinson, 2024; Han and Miao, 2024; He et al., 2022; Zhou and Pichersky, 2020b). Among these metabolites, terpenoids constitute the largest and most structurally diverse class. Terpene synthases (TPSs) constitute the key enzyme family responsible for generating terpenoid structural diversity (Dixon and Dickinson, 2024; Wang et al., 2025). The biosynthesis of terpenes originates from two compartmentalized pathways in plants: the plastidial MEP pathway and the cytosolic mevalonate (MVA) pathway, both of which sequentially generate the universal C_5_ precursors isopentenyl diphosphate (IPP) and dimethylallyl diphosphate (DMAPP) (Conart et al., 2023; Song et al., 2023; Xue et al., 2022; Zheng et al., 2017; Zhou and Pichersky, 2020a). Through successive condensations catalyzed by short-chain prenyltransferases, these precursors give rise to C_10_ GPP or NPP, C_15_ FPP, and C_20_ geranylgeranyl diphosphate (GGPP) (Yang et al., 2025a). These prenyl diphosphates are subsequently converted into an enormous diversity of terpenoid skeletons through a series of rearrangements and cyclization reactions catalyzed by terpene synthases. The copy number, subcellular localization, and catalytic specificity of TPSs are therefore key determinants of terpenoid diversity within a given plant species (Bao et al., 2023; He et al., 2022; Yan et al., 2023).

As a medium-sized gene families, TPS genes have undergone extensive duplication, neofunctionalization, and subfunctionalization throughout plant evolution (Chen et al., 2020b; Eljounaidi et al., 2025; Li et al., 2024a; Zhou and Pichersky, 2020b). Comparative genomic studies have revealed that lineage-specific TPS expansions frequently correlate with ecological specialization and the accumulation of species-specific metabolites (Ding et al., 2020; Hendrickson et al., 2024; Kumeta and Ito, 2016; Zhan et al., 2021). For instance, members of the *Asteraceae* harbor more than 1,700 TPS genes, predominantly from the TPS-a and TPS-b clades, which underpin the biosynthesis of pharmacologically important sesquiterpenes such as artemisinin and caryophyllene (Yang et al., 2025b). Gene duplication, whole-genome duplication (WGD), and the formation of biosynthetic gene clusters (BGCs) are recognized as major drivers shaping TPS repertoires and promoting functional diversification across plant lineages (Sun et al., 2023, Sun et al., 2022).

Soybean (*Glycine max*), one of the most important crops globally, functions not only as a major source of plant-derived protein and vegetable oil but also as a rich reservoir of diverse secondary metabolites with both ecological and nutritional importance (Gao et al., 2021; Kim et al., 2023; Zhang et al., 2018, Zhang et al., 2017). Soybean contains a variety of bioactive secondary metabolites, including flavonoids such as anthocyanins and isoflavones. In addition, terpenoid, particularly monoterpenes and sesquiterpenes, play important roles in plant defense and ecological communication between plants and other organisms (Du et al., 2020; Ha et al., 2018; Tu et al., 2025; Zhou and Pichersky, 2020b). For instance, Del Rosario et al. (1984) first identified the monoterpene α-pinene in wild soybean accessions. Subsequent studies indicated that (E)-β-ocimene and linalool may play important roles in insect resistance among specific soybean cultivars (Liu et al., 1989). Moreover, (E, E)-α-farnesene has been reported as one of the most variable compounds between damaged and undamaged soybean plants, suggesting a role in herbivore-induced defense responses (Lin et al., 2017). Our previous work identified GmOCS (GmTPS3) as the enzyme responsible for (E)-β-ocimene biosynthesis (Han et al., 2023); however, the GmTPS genes mediating linalool formation have yet to be functionally characterized.

Although recent findings suggest that soybean TPS genes play central roles in ecological adaptation, reproductive development, and stress responses (Han et al., 2023; Li et al., 2024b; Lin et al., 2017; Liu et al., 2014; Zhang et al., 2013), key questions remain unresolved: (i) how TPS gene repertoires vary among soybean accessions; (ii) what evolutionary pressures shape the divergence of TPS subfamilies; (iii) how structural variations influence TPS gene models; and (iv) what biochemical functions and ecological roles are associated with lineage-specific TPS members. Addressing these questions is essential to elucidate the genetic and evolutionary basis of terpenoid biosynthesis in soybean, as well as its contributions to plant defense, crop resilience, and metabolic engineering. The recent availability of high-quality soybean pan-genomes now enables systematic exploration of TPS diversity at the population level, capturing presence/absence variation and structural polymorphisms invisible to single-reference approaches (Liu et al., 2020).

To clarify the genetic diversity, evolutionary history, and enzymatic mechanisms underlying terpene metabolism in soybean, this study first employed pan-genome mining to identify TPS loci conserved across soybean accessions. Based on this framework, we then focused on previously uncharacterized genes potentially associated with soybean defense and performed targeted functional characterization. Here, we report for the first time the identification of *GmTPS20* as the terpene synthase gene responsible for linalool biosynthesis. These findings not only elucidate the diversification mechanisms of TPSs in legumes but also provide valuable molecular resources for future metabolic engineering aimed at enhancing terpenoid-based defense and value-added traits in soybean.

## 2 Materials and methods

### 2.1 Plant materials and growth conditions

Soybean (*Glycine max* cv. Williams 82) plants were grown in a controlled-environment greenhouse under a 16 h light/8 h dark photoperiod at 22–24 °C, from March to May 2024, corresponding to the spring growing season. *Arabidopsis thaliana* (Col-0) and *Nicotiana benthamiana* (provided by the laboratory of Professor Xiang Gao, Northeast Normal University) were maintained under identical conditions at 22 °C with a 16 h/8 h light/dark cycle.

### 2.2 Identification of TPS genes

For soybean pan-genome analyses, predicted protein-coding sequences were retrieved from a published soybean pan-genome dataset comprising 27 genome assemblies, including the reference genome of Glycine max cv. Williams 82 (Wm82.a2.v1), obtained from Phytozome (https://phytozome-next.jgi.doe.gov), accessed on 12 October 2024. The additional 26 soybean genome assemblies and corresponding annotations were obtained from the published soybean pan-genome resource (https://ngdc.cncb.ac.cn/bioproject/browse/PRJCA002030), accessed on 12 October 2024.

Protein sequences from the soybean pan-genome were screened using HMMER v3.3 with Pfam hidden Markov models (HMMs) corresponding to the TPS N-terminal (PF01397) and C-terminal (PF03936) domains, with an E-value cutoff of 1×e^-5^. Candidate proteins were further validated for conserved TPS domains using the SMART (https://smart.embl.de/smart/change_mode.cgi) and the NCBI Conserved Domain Database (https://www.ncbi.nlm.nih.gov/Structure/cdd/wrpsb.cgi?SEQUENCE), both accessed on 12 October 2024. Redundant isoforms were removed, retaining the longest coding sequence for each locus.

### 2.3 Definition of gene presence/absence categories across 27 soybean assemblies

We classified genes in the pan-genome into four distinct categories based on gene presence/absence variation across 27 soybean genome assemblies, employing the following frequency thresholds:

1. Core genes: Present in ≥99% of all accessions analyzed. This stringent criterion accommodates rare assembly gaps and annotation inconsistencies, thereby minimizing the misclassification of biologically indispensable single-copy genes.
2. Near-Core genes: Present in ≥95% and <99% of accessions. These genes are broadly conserved across the soybean germplasm but have undergone sporadic loss within a limited subset of lineages.
3. Variable genes: Present in ≥5% and <95% of accessions. This fraction constitutes the majority of dispensable genomic content and represents the flexible, adaptive gene pool of the species.
4. Private genes: Present in <5% of accessions. These genes are typically restricted to a single accession or a highly constrained genetic background.

### 2.4 Phylogenetic analysis

Soybean and Arabidopsis TPS protein sequences were aligned using the online Clustal Omega service (http://www.ebi.ac.uk/Tools/msa/clustalo/) with default parameters for sequence alignment. The alignment was subsequently processed by MEGA 7 to construct a neighbor-joining tree with 1000 bootstraps (Kumar et al., 2018). Sequences with the following GenBank accession numbers were used in either phylogeny or alignment analysis: AtTPS10 (ACF41947), AtTPS14 (NP001185286), AtTPS21 (NP001190374). The resulting trees were visualized with iToL (https://itol.embl.de/), accessed on 12 October 2024.

### 2.5 Selection pressure analysis

Orthologous coding sequences of each *GmTPS* gene across 27 soybean genomes were codon-aligned using PAL2NAL (Suyama et al., 2006). Pairwise nonsynonymous (Ka) and synonymous (Ks) substitution rates were calculated with KaKs_Calculator 2.0 using the YN00 model. The median Ka/Ks ratio was reported for each gene. Genes with Ka/Ks < 1 were considered to be under purifying selection, whereas those with Ka/Ks > 1 were inferred to be under positive selection.

### 2.6 Gene structure and conserved motif analysis

Exon-intron structures of *GmTPS* genes were visualized using TBtools (Chen et al., 2020a). Conserved motifs were identified with MEME Suite v5 using the zoops model, with the number of motifs set to 10 and widths ranging from 6 to 50 amino acids (Bailey et al., 2009).

### 2.7 Subcellular localization

The coding sequences of *GmTPS15* and *GmTPS20*, excluding the stop codon, were cloned in-frame upstream of GFP into the pUC19-GFP vector, in which the GFP expression cassette is driven by the CaMV 35S promoter. The resulting constructs were introduced into *Arabidopsis* mesophyll protoplasts via polyethylene glycol (PEG)-mediated transfection, as described previously (Díaz and Koop, 2014; Li et al., 2020). Following overnight incubation, GFP fluorescence was observed under a confocal laser scanning microscope (excitation at 488 nm; emission at 500–540 nm). Chlorophyll autofluorescence was detected at 650–750 nm to evaluate plastid localization.

### 2.8 Heterologous expression in *N. benthamiana*

Full-length coding sequences of *GmTPS15* and *GmTPS20* were cloned into the pEAQ-HT vector under the control of the CaMV 35S promoter (Sainsbury et al., 2009). *Agrobacterium tumefaciens* strain GV3101 cultures (OD_600_ = 0.6), pre-induced with 150 µM acetosyringone, were infiltrated into *N. benthamiana* leaves using a needleless syringe. Plants were maintained at 22–24 °C under high humidity, and leaves were collected at 4 days post-infiltration (dpi) for volatile compound analysis.

### 2.9 Recombinant protein expression and purification

*GmTPS15* and *GmTPS20* coding sequences were cloned into His-tagged bacterial expression vector pET-32a (+) and pMal-c2x, respectively, and subsequently transformed into *Escherichia coli* BL21 (DE3) cells. Cultures were induced at an OD_600_ of approximately 0.6 with 0.3 mM IPTG and incubated at 16 °C for 16 h. Recombinant proteins were purified using Ni-NTA affinity chromatography and subsequently buffer-exchanged into assay buffer (50 mM Tris-HCl, pH 7.5; 10 mM MgCl_2_; 10% glycerol). Protein concentrations were quantified using the Bradford assay.

### 2.10 *In vitro* enzyme assays

Enzyme assays (200 µL total volume) were performed in a reaction buffer containing 50 mM Tris-HCl (pH 7.5), 10 mM MgCl_2_, 5 mM DTT, 10% glycerol, 20 µg of purified enzyme, and 20–50 µM substrate (GPP, NPP, (E,E)-FPP, or (Z,Z)-FPP). Reactions were overlaid with hexane and incubated at 30 °C for 1 h. Volatile products were extracted into the organic phase and subsequently analyzed by GC-MS.

### 2.11 GC-MS analysis of volatiles

Headspace SPME-GC/MS was employed to examine the volatile terpenes following our earlier studies (Bao et al., 2020; Yang et al., 2022). Briefly, a solid phase microextraction fiber assembly (57329-U, Sigma-Aldrich, St. Louis, MO, USA) was used to collect volatiles at 22 °C for 2 h. The volatiles captured by the fibers were subsequently thermally desorbed and analyzed by an Agilent 5975-6890N GC-MS apparatus (Agilent Technologies, Santa Clara, CA, USA) equipped with an HP-1MS fused-silica capillary column. Volatile terpenes were identified by comparing the mass spectra and retention times with those of standard samples or compounds deposited in the NIST 2008 mass spectra library. The relative contents of volatile terpenes were quantified based on a linalool standard curve, as linalool was the major volatile terpene released from soybean leaves.

### 2.12 Bioinformatic analysis of TPS-substrate binding

Three-dimensional structure of the TPS proteins were predicted using AlphaFold3. Model confidence was assessed using per-residue confidence scores (pLDDT) extracted from the predicted structures. Subsequently, the predicted TPS structure was superimposed onto the previously resolved TPS complex with FPP (PDB ID: 3g4f) using the Molecular Operating Environment (MOE) software. In the case of TPS utilizing GPP as the substrate, the GPP molecule was first aligned to FPP based on the phosphorus atoms and the bridging oxygen atom before proceeding with the docking procedure. For structural alignment, residues within 4.5 Å of the FPP molecule and the associated magnesium ion were selected. Following superposition, molecular docking was performed in MOE with the receptor set to the induced-fit mode.

### 2.13 Accession numbers

Genome assemblies and annotations for the 26 soybean accessions were obtained from the published soybean pan-genome resource under BioProject accession PRJCA002030 (Liu et al., 2020). RNA-seq reads used for tissue-expression analyses were retrieved from the corresponding public datasets. In addition, RNA-seq data related to aphid infestation have been deposited in the NCBI SRA under BioProject accession PRJNA594515 (Yao et al., 2020).

## 3 Results

### 3.1 Identification and phylogenetic classification of soybean TPS genes

**Figure 1:**
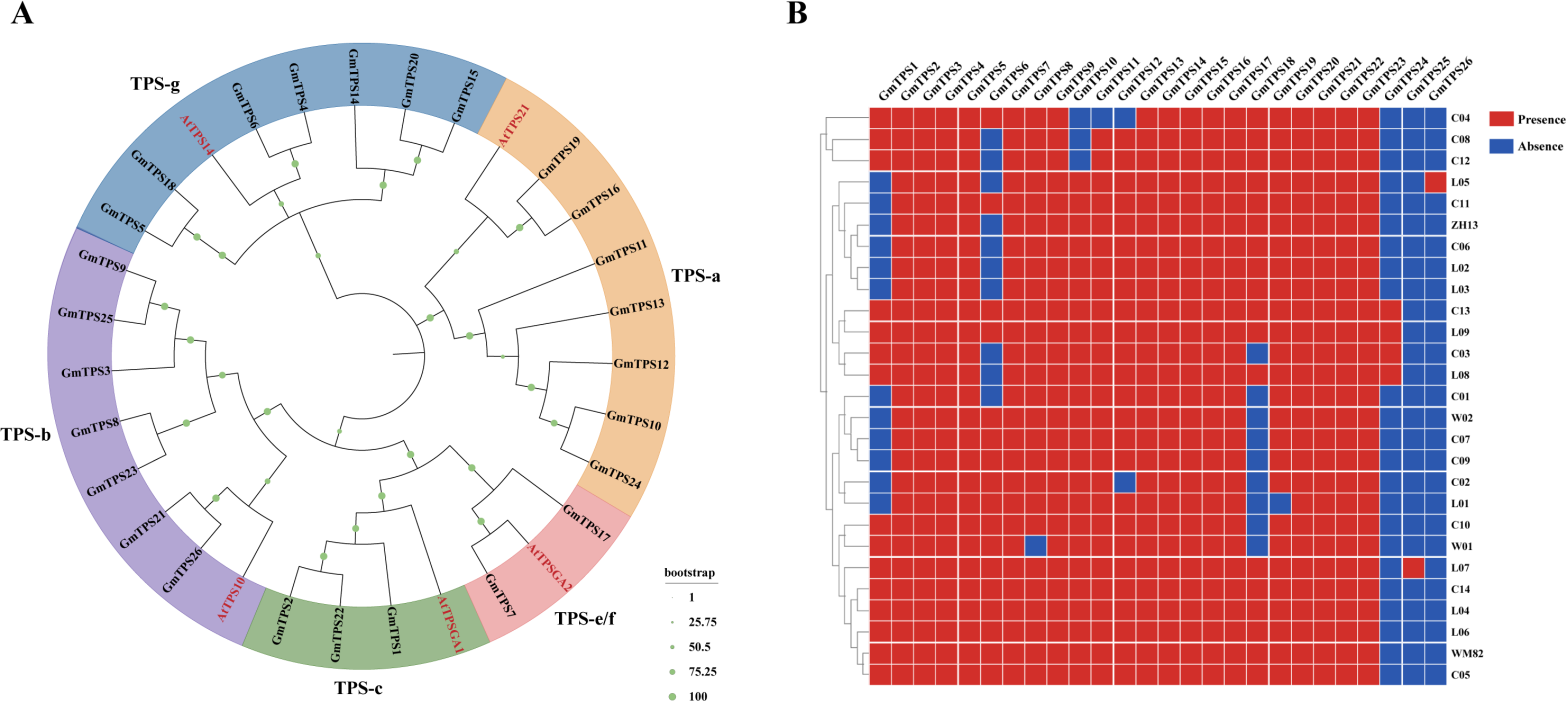
Phylogenetic relationships and pan-genome classification of terpene synthase (TPS) genes in soybean. **(A)** Phylogenetic tree of TPS proteins from soybean and *Arabidopsis*. Soybean TPS genes were classified into five subfamilies (TPS-a, TPS-b, TPS-c, TPS-e/f, and TPS-g) based on *Arabidopsis* TPSs. **(B)** Pan-genome distribution of 26 soybean TPS genes across 27 genomes. TPS members were categorized into core, near-core, variable, and private genes. Core TPS genes were conserved across most genomes, whereas *GmTPS24, GmTPS25*, and *GmTPS26* exhibited the highest deletion frequency, and *GmTPS1*, *GmTPS6*, and *GmTPS18* showed cultivar-specific losses.

Using pan-genome mining, a total of 26 TPS genes were identified across the soybean pan-genome (**Supplementary Table 1**). A maximum-likelihood phylogenetic tree constructed from soybean and *Arabidopsis* TPS proteins divided the soybean TPS family into five clades: TPS-a, TPS-b, TPS-c, TPS-e/f, and TPS-g (**Figure 1A**). Pan-genome profiling further categorized the 26 soybean TPS homologs into 15 core, 4 near-core, 5 variable, and 2 private genes (**Figure 1B**). Notably, *GmTPS24*, *GmTPS25*, and *GmTPS26* were absent in 26 of 27 accessions, including Wm82, representing the highest loss frequency among the variable genes. In contrast, *GmTPS1*, *GmTPS6*, and *GmTPS18* displayed cultivar-specific losses. This uneven distribution suggests lineage-specific retention and loss within the soybean TPS family, with core members exhibiting greater evolutionary stability across soybean accessions.

### 3.2 Selection pressure analysis of soybean TPS genes

**Figure 2:**
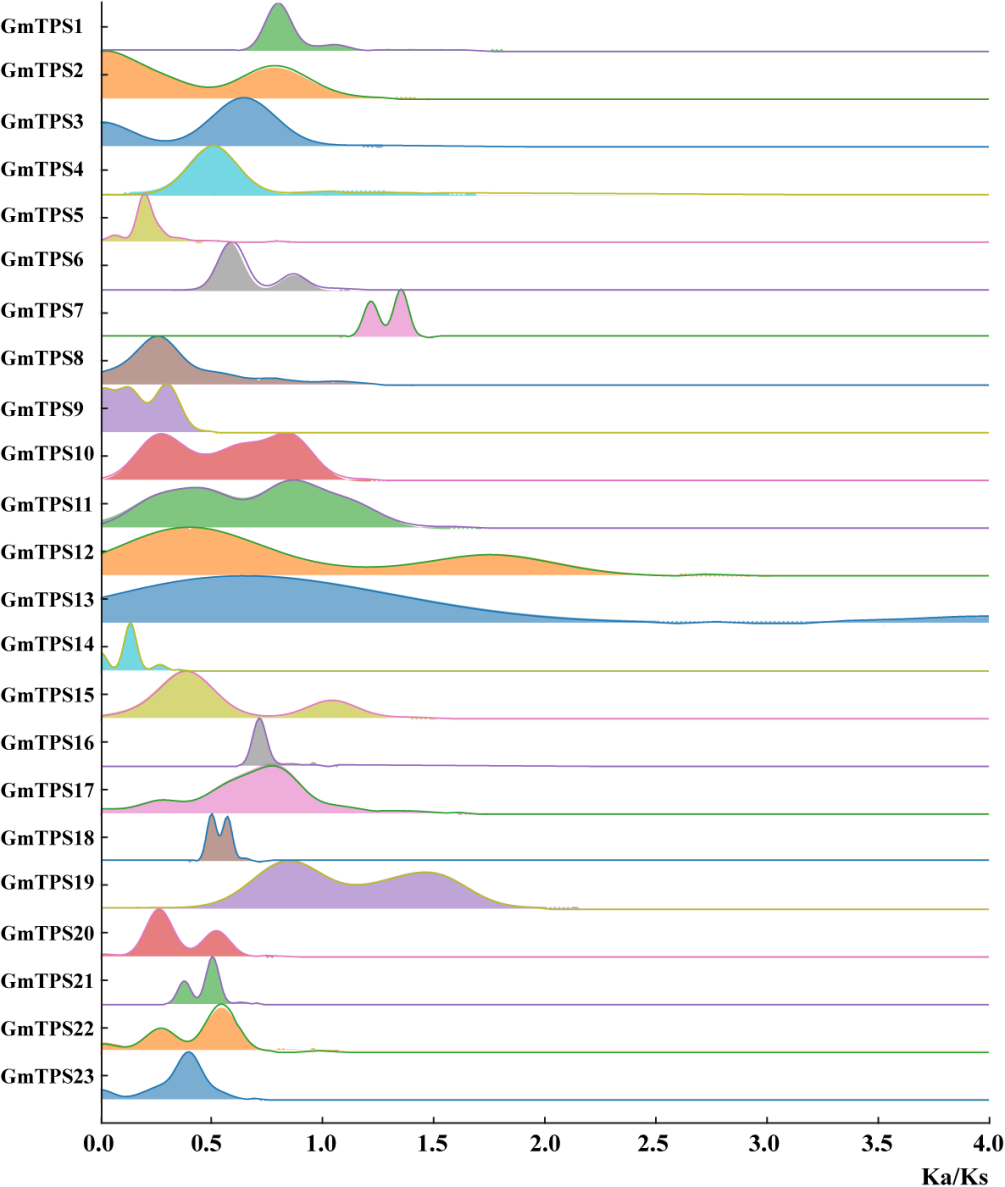
Selection pressure analysis of soybean TPS genes. Distribution of Ka/Ks ratios of *GmTPS* genes across 27 soybean genomes. Most TPS members exhibited Ka/Ks < 1, indicating strong purifying selection, whereas *GmTPS5*, *GmTPS14*, *GmTPS18*, *GmTPS20*, and *GmTPS21* showed particularly low values, suggesting essential biological roles constrained by negative selection. In contrast, *GmTPS7* and some accession-specific orthologs of *GmTPS11, GmTPS12, GmTPS13, GmTPS15*, and *GmTPS19* displayed Ka/Ks > 1, suggesting possible positive selection during soybean diversification.

To explore the selection pressure on GmTPS genes, we calculated the Ka/Ks value for each TPS gene based on the gene sequences in the 27 soybean genomes. Most genes (21 out of 26) exhibited Ka/Ks values below 1, indicative of strong purifying selection (**Figure 2**). A subset of genes, including *GmTPS5*, *GmTPS14*, *GmTPS18*, *GmTPS20*, and *GmTPS21*, displayed particularly low Ka/Ks ratios, suggesting strong functional constraints and essential biological roles. In contrast, *GmTPS7 and* some accession-specific orthologs of *GmTPS11, GmTPS12, GmTPS13, GmTPS15*, and *GmTPS19* showed Ka/Ks > 1, reflecting positive selection during soybean diversification. Collectively, the evidence indicates differential selection acting on discrete members of the GmTPS family.

### 3.3 Candidate gene selection within the GmTPS family based on functional and expression evidence

**Figure 3:**
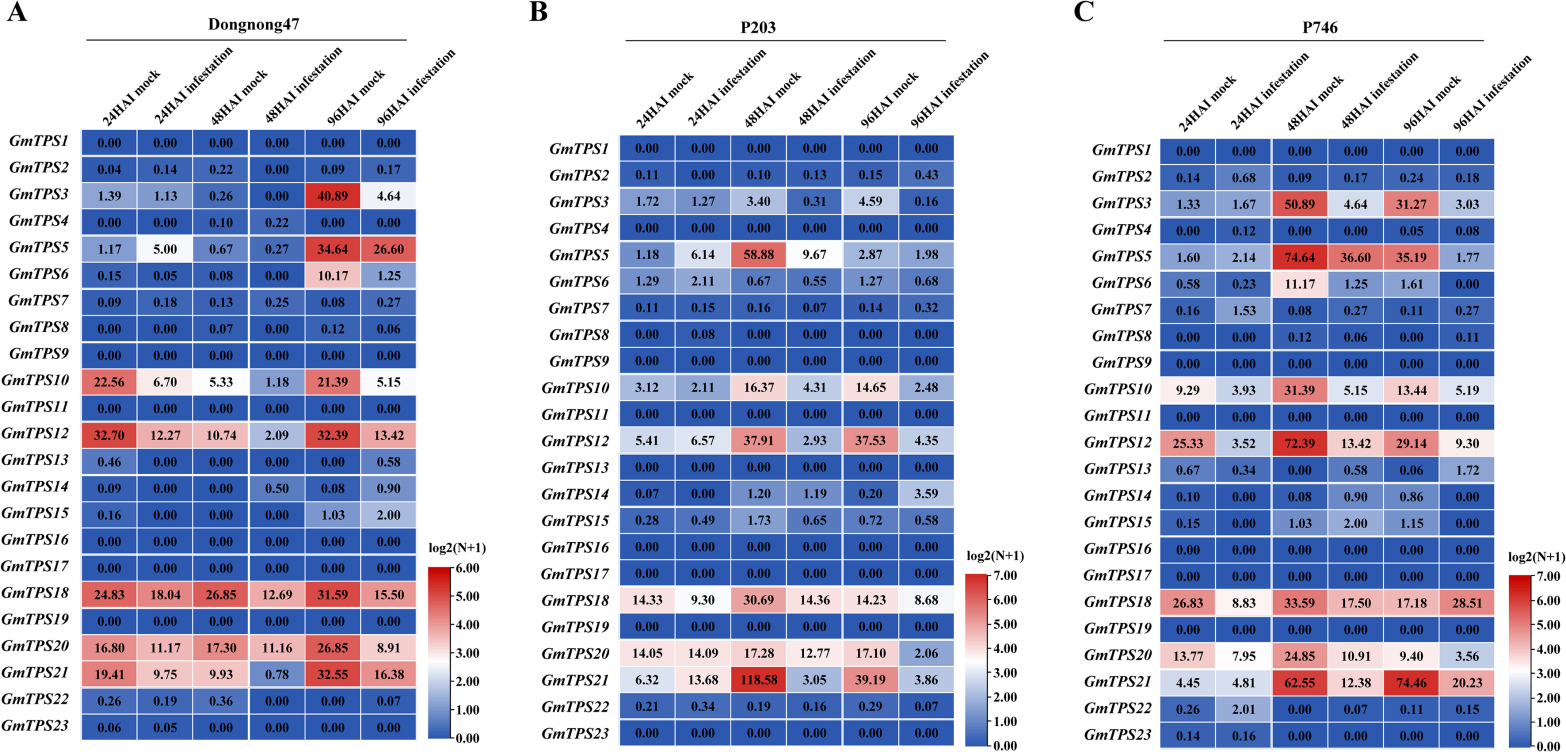
Differentially expressed GmTPS genes in response to aphid infestation. **(A–C)** Heatmaps showing the expression levels of *GmTPS* genes in soybean lines Dongnong47, P203, and P746, respectively. Expression values (FPKM) were derived from previously published RNA-seq data (Yao et al., 2020). The FPKM values were log2-transformed and visualized using TBtools (Heatmap Illustrator).

Among the 26 identified members of the GmTPS family in the soybean genome (**Figure 1**), the functions of several genes have been elucidated, such as *GmTPS5* and *GmTPS18* (*GmNES*), which encode geraniol synthase and nerol synthase, respectively, and confer resistance against cotton leafworms (*Spodoptera litura*) in transgenic tobacco plants (Liu et al., 2014; Zhang et al., 2013). In addition, *GmTPS21* (*GmAFS*) encodes an α-farnesene synthase implicated in defense responses against nematodes and insect pests (Lin et al., 2017). To further clarify the putative roles of each TPS gene, we examined their differential expression patterns under aphid feeding conditions using transcriptomic data. Aphid infestation induced significant changes in the expression of *GmTPS3*, *GmTPS5*, *GmTPS10*, *GmTPS12*, *GmTPS18*, *GmTPS20*, and *GmTPS21* across different time points and across the three soybean genotypes Dongnong 47, P203, and P746 (**Figure 3**), suggesting that these genes may participate in conserved transcriptional responses to herbivory. Among these, *GmTPS3*, *GmTPS5*, *GmTPS18*, and *GmTPS21* have been functionally characterized in previous studies. Given that aphid feeding is known to activate jasmonic acid (JA)-mediated signaling, and our previous work established that MeJA treatment induces significant linalool production accompanied by a pronounced upregulation of *GmTPS20* (Han et al., 2023), we prioritized this uncharacterized core gene member for subsequent functional investigation. Among the identified TPS genes, *GmTPS15* resides within the same phylogenetic clade as *GmTPS20* and exhibits a high degree of sequence conservation, sharing 91.06% nucleotide identity and 94.31% amino acid similarity with *GmTPS20* (**Figure 1A**). As the closest paralog of *GmTPS20* within the soybean TPS-g subfamily, *GmTPS15* was therefore investigated in parallel to enable a direct comparative assessment of function and catalytic mechanism.

### 3.4 Structural variations and motif divergence of GmTPS15 and GmTPS20

**Figure 4:**
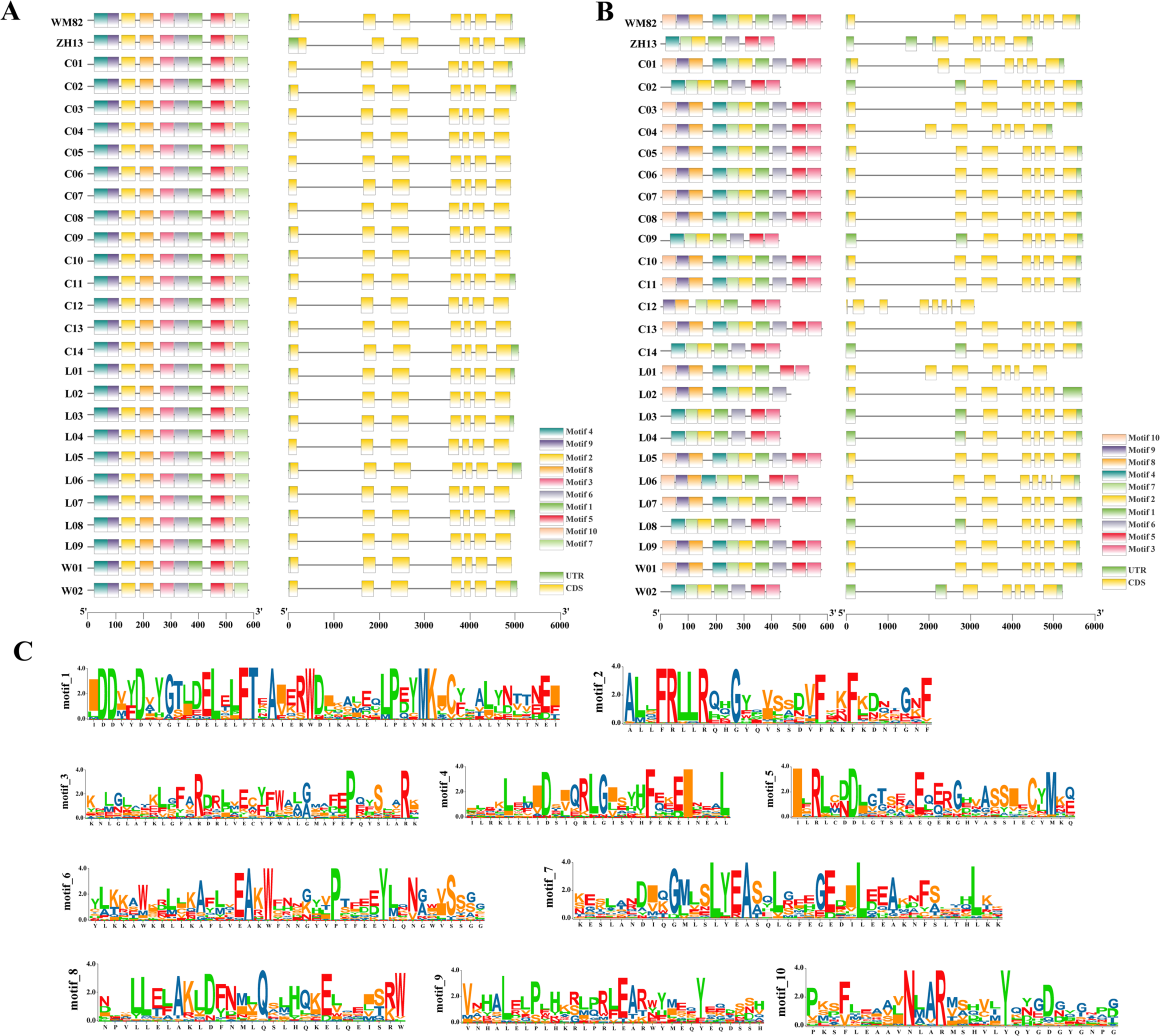
Structural variation and motif divergence of *GmTPS15* and *GmTPS20*. **(A)** Gene structure and conserved motif organization of *GmTPS15* across 27 soybean accessions. **(B)** Gene structure and conserved motif organization of *GmTPS20* across 27 soybean accessions. **(C)** Amino acid sequences of the ten conserved motifs identified in *GmTPS15* and *GmTPS20*.

Structural variation analysis of GmTPS15 and GmTPS20 across 27 soybean accessions revealed differences in motif architecture. All ten conserved motifs were present in *GmTPS15* across all accessions examined, whereas motif 10 of GmTPS20 was absent in nine accessions (e.g., ZH13, C02, C12) (**Figures 4A, B**). Such truncation events suggest that variation in gene architecture may drive functional diversification or pseudogenization. Furthermore, sequence alignment revealed that even when motifs are present, the amino acid composition within these conserved motifs varies among *GmTPS* genes (**Figure 4C**). The imperfect conservation of these motifs indicates that genetic structural variations have impacted the conserved domains of different *GmTPS* genes, potentially contributing to the generation of divergent or atypical gene family members.

### 3.5 Tissue-specific expression patterns and subcellular localization

**Figure 5:**
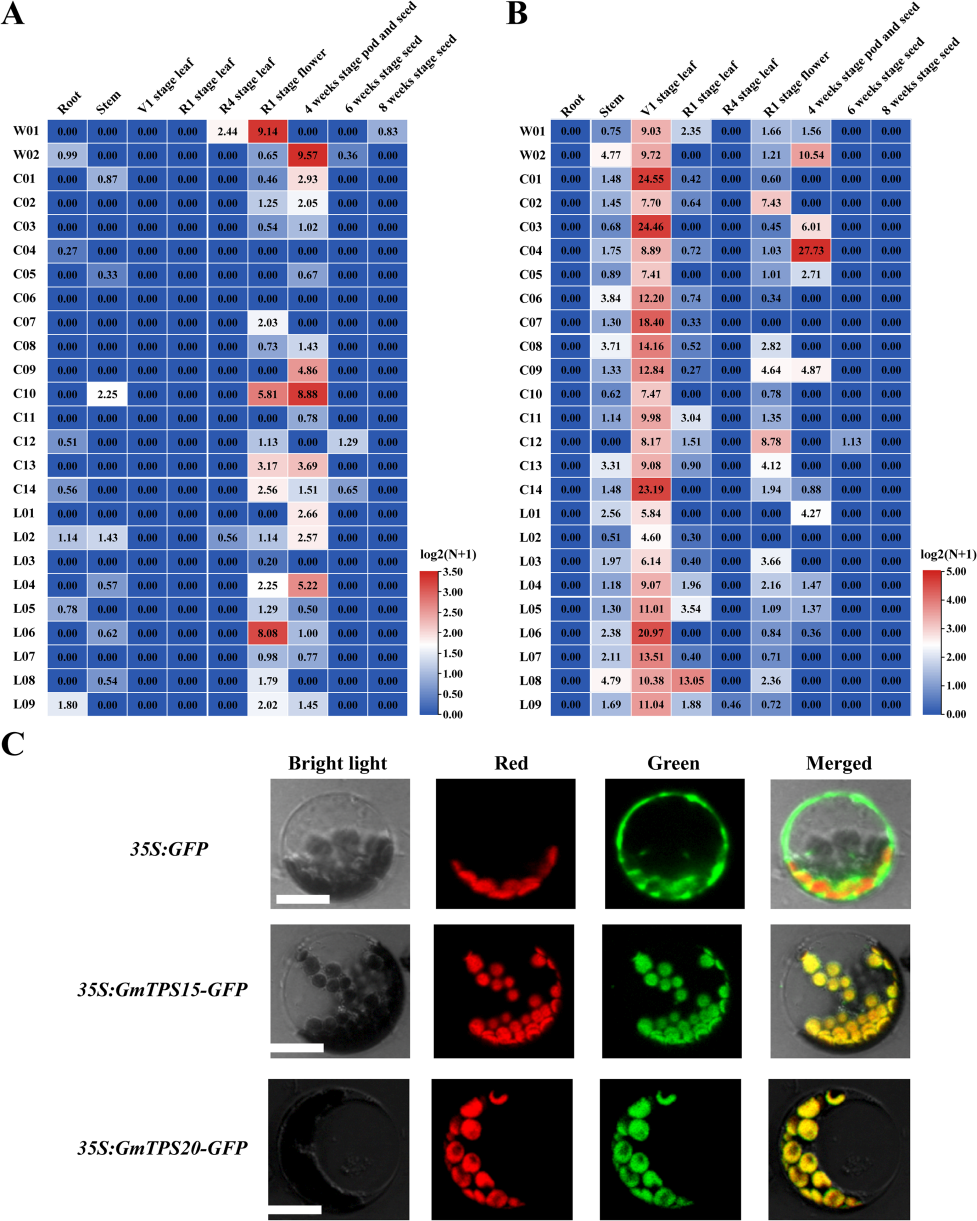
Tissue-specific expression patterns and Subcellular localization of soybean terpene synthase genes *GmTPS15* and *GmTPS20*. **(A)** Expression profile of *GmTPS15* across multiple tissues and developmental stages based on RNA-seq data. **(B)** Transcripts of *GmTPS20* in different tissues or organs of soybean. The data were calculated with earlier published RNA-seq data (Liu et al., 2020). The FPKM values were log2-transformed and visualized using TBtools (Heatmap Illustrator). **(C)** Subcellular localization of GmTPS15 and GmTPS20 in *Arabidopsis* protoplasts. Green indicates GFP fluorescence, red indicates chlorophyll autofluorescence, brightfield indicates the brightfield image, and merged indicates the overlay of all channels. Scale bar, 25 µm.

RNA-seq expression profiling revealed distinct expression patterns between *GmTPS15* and *GmTPS20*. *GmTPS15* showed no detectable expression in roots, stems, leaves, or seeds but was specifically upregulated in flowers at the R1 stage and in pods at four weeks in a subset of accessions (**Figure 5A**). In contrast, *GmTPS20* exhibited broad expression across multiple tissues, with significantly higher transcript levels in young leaves at the V1 stage (**Figure 5B**). To determine subcellular localization, GFP fusion constructs of *GmTPS15* and *GmTPS20* were transiently expressed in *Arabidopsis* protoplasts (**Supplementary Table 2**). Confocal microscopy revealed that *GmTPS15* and *GmTPS20* were targeted to chloroplasts (**Figure 5C**). These observations are consistent with their predicted biochemical functions: monoterpene biosynthesis takes place in chloroplasts.

### 3.6 Catalytic function of GmTPS15 and GmTPS20

**Figure 6:**
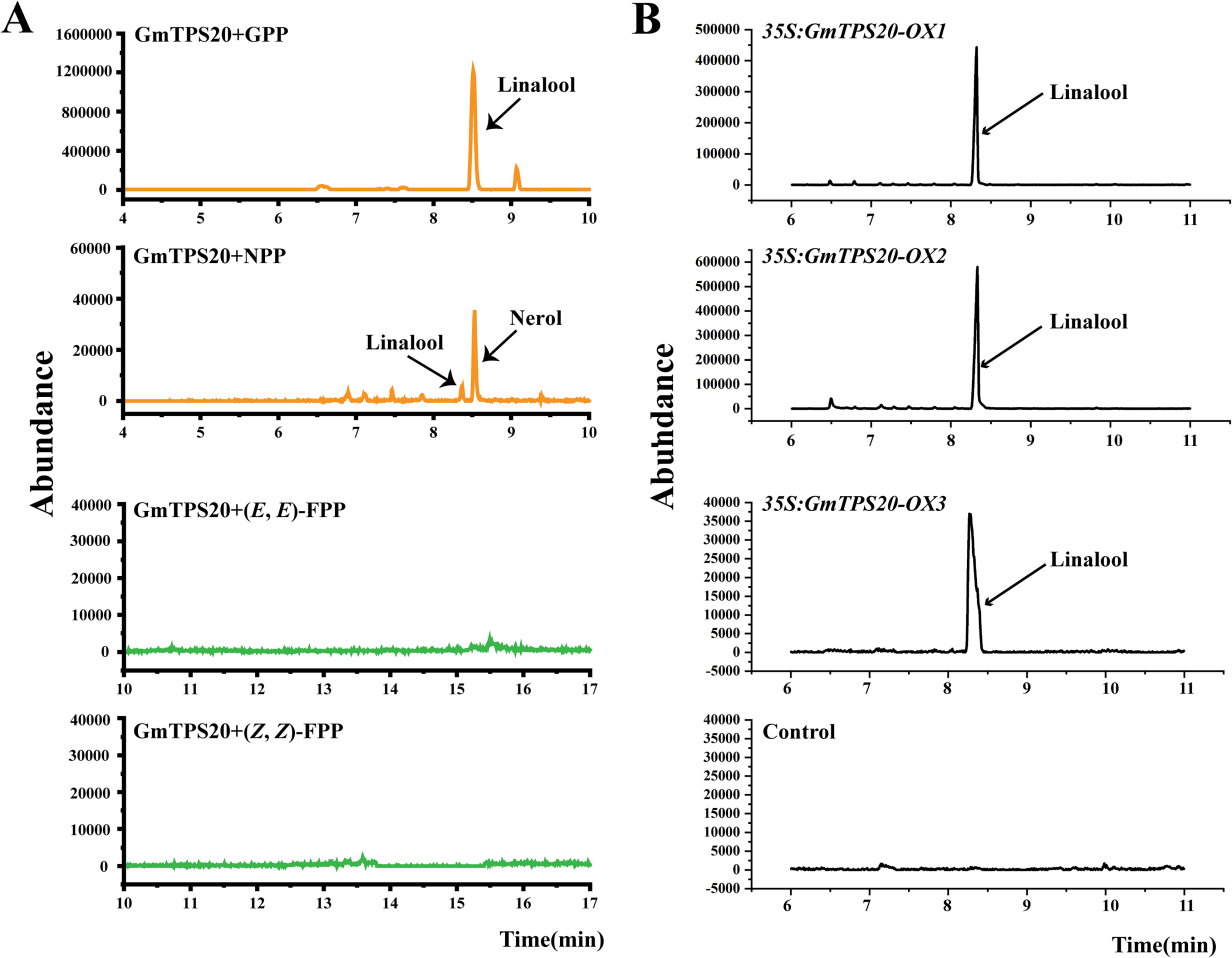
*In vitro* and *in vivo* characterization of *GmTPS20*. **(A)** *In vitro* enzymatic analysis of GmTPS20 using four acyclic prenyl diphosphate substrates. The x-axis represents retention time, and the y-axis represents product abundance. **(B)** Volatile terpenes released from (N) benthamiana leaves transiently overexpressing *GmTPS20*. *Agrobacterium* harboring *GmTPS20* in the pEAQ-HT backbone was infiltrated into *N. benthamiana* leaves. The leaves were sampled and analyzed by GC-MS analysis. *N. benthamiana* leaves infiltrated by *Agrobacterium* containing empty pEAQ-HT vector were used as the control.

The GmTPS15 and GmTPS20 proteins were prepared through prokaryotic expression and purification (**Supplementary Figure 1**). Subsequently, enzymatic assays were performed with four acyclic prenyl diphosphate substrates: GPP, NPP, (E, E)-FPP, and (Z, Z)-FPP. The results showed that GmTPS15 did not produce any detectable volatile compounds when incubated with the four tested substrates (**Supplementary Figures S2**–**S4A**). To further evaluate the function of GmTPS15, we constructed a transient expression vector harboring GmTPS15 driven by CaMV 35S promoter and introduced it into *A. tumefaciens* GV3101 which was subsequently infiltrated into *N. benthamiana* leaves. Consistent with the *in vitro* assays, no volatile products were detected from *GmTPS15*-expressing *N. benthamiana* leaves at 4 days post-infiltration (**Supplementary Figures S4B**; **S5**). In contrast, *GmTPS20* converted GPP exclusively into linalool and converted NPP into both nerol and linalool, but showed no detectable activity with (E,E)-FPP, and (Z,Z)-FPP substrates (**Figure 6A**). Transient expression in *N. benthamiana* leaves mainly released linalool (**Figure 6B**; **Supplementary Figure S6**), validating the result obtained from the *in vitro* enzymatic assays. Collectively, these observations demonstrate that GmTPS15 has lost catalytic function, while GmTPS20 retains strict substrate specificity as a dedicated monoterpene synthase.

### 3.7 Structural basis for substrate recognition by GmTPS15 and GmTPS20

**Figure 7:**
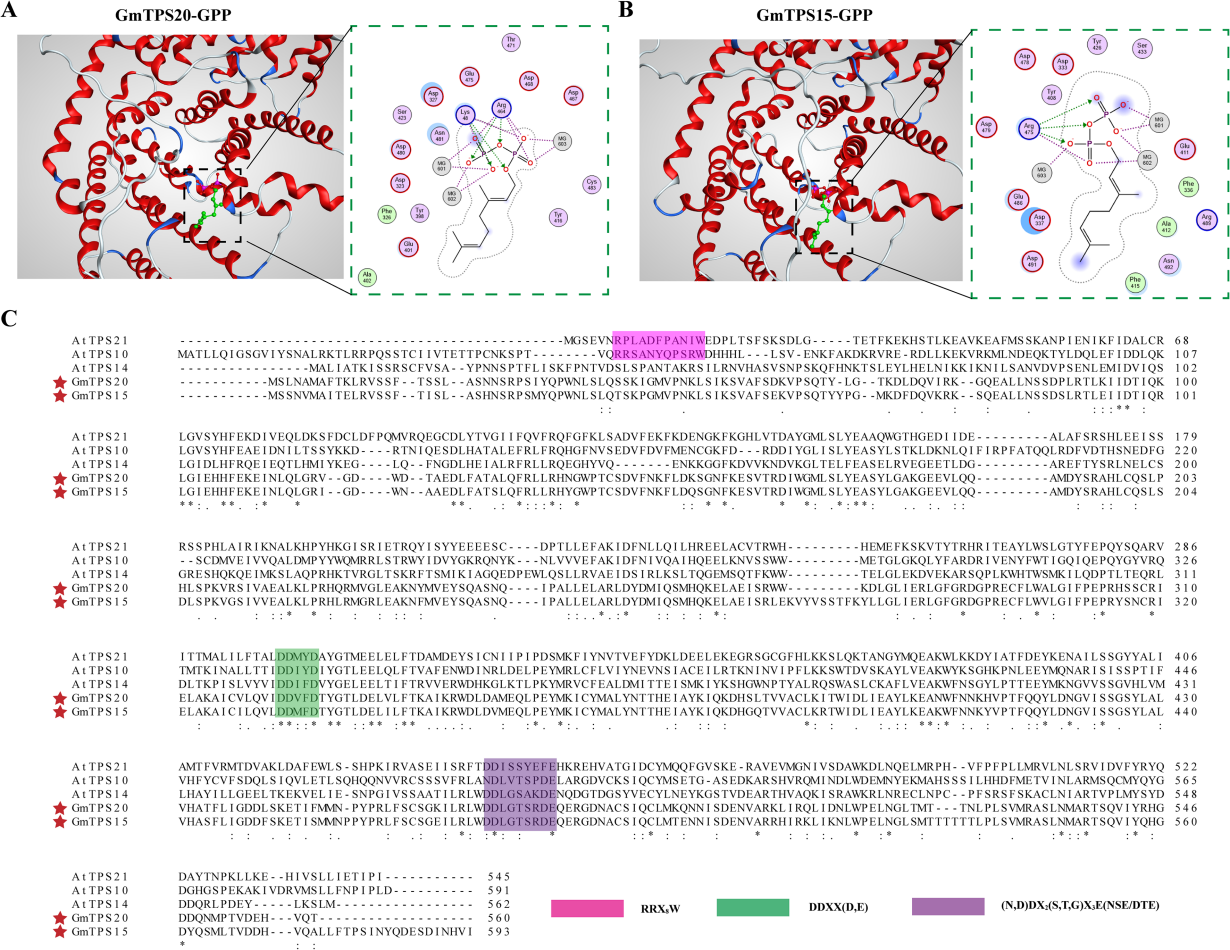
TPS-GPP docking and conserved-site mapping. **(A)** GmTPS20-GPP molecular docking. Color key for 2D interaction maps: grey outlines indicate polar residues, red outlines indicate acidic residues, blue outlines indicate basic residues, and green circles indicate proline. **(B)** GmTPS15-GPP molecular docking. **(C)** Amino acid sequence alignment of GmTPS15 and GmTPS20 proteins. Conserved amino acid residues are highlighted.

To further elucidate the catalytic mechanisms of *GmTPS15* and *GmTPS20*, we conducted structural modeling, molecular docking, and amino acid sequence comparisons. The predicted structures showed high model confidence, with AlphaFold3 pLDDT values exceeding 85. Molecular docking further yielded binding energies below −4.0 kcal/mol (**Supplementary Table 3**). As shown in **Figures 7A, B**, GPP adopts a catalytically competent pose in both GmTPS20 and GmTPS15, with the diphosphate moiety oriented toward the metal-binding cavity and anchored by the conserved DDxxD and NSE/DTE motifs, while the isoprenoid chain extends along a hydrophobic groove. Comparative analysis of residues surrounding the docked substrate GPP revealed that GmTPS20 and GmTPS15 differ at only one position within the immediate binding pocket, corresponding to F415 in GmTPS15 and Y416 in GmTPS20. In addition, GmTPS20 contains two polar residues, K48 and R464, that are positioned to directly interact with the substrate, whereas GmTPS15 contains only one corresponding residue (R475), resulting in one fewer predicted substrate-contacting residue. Furthermore, although both enzymes contain five Asp residues within the binding pocket, four Asp residues in GmTPS20 are positioned around the diphosphate moiety of GPP, representing one additional Asp residue compared with GmTPS15. In summary, compared with GmTPS15, the GmTPS20-GPP complex is predicted to display a denser network of hydrogen-bond/ionic contacts to the diphosphate and a deeper, more constricted hydrophobic pocket. At the same time, we also examined four TPS genes previously identified with specific functions in soybean, whose complexes were likewise predicted to form deeper and narrower hydrophobic pockets (**Supplementary Figure 7**). In contrast, GmTPS15 presents a more open cavity with weaker polar constraints on the diphosphate group. These structural features may impair productive substrate anchoring and help explain the absence of detectable catalytic activity under the tested conditions. Detailed amino acid alignment (**Figure 7C**) indicated that *GmTPS15* and *GmTPS20* contain the Mg^2+^ or Mn^2+^ cofactor-binding-related DDxx(D/E) and (N,D)Dxx(S,T,G)xxxE(NSE/DTE) motifs, without terpene-cyclization-related RR(x)8W motif. Given that GmTPS20 predominantly produces the acyclic monoterpenoid linalool, the absence of the canonical RR(x)8W motif is consistent with previous reports (Yan et al., 2023; Zhan et al., 2021). Pocket-lining substitutions at hydrophobic/volume-shaping positions differentiate the two active sites and are consistent with the observed cavity geometry. Together, these features suggest that GmTPS20 affords tighter substrate anchoring and stricter substrate orientation, predicting higher product selectivity, whereas GmTPS15 provides a more open cavity with weaker polar constraints, which may compromise productive substrate positioning and thereby contribute to its loss of detectable catalytic activity.

## 4 Discussion

In this study, we integrated soybean pan-genome analysis with evolutionary, expression, biochemical, and structural approaches to investigate functional divergence within the TPS gene family. A total of 26 TPS loci were identified across multiple soybean genomes, revealing pronounced heterogeneity in gene conservation and presence-absence variation. Among these genes, GmTPS20 emerged as a conserved core member under strong purifying selection and showed inducible expression in response to aphid infestation and methyl jasmonate treatment. Functional assays suggested that GmTPS20 encodes a plastid-localized monoterpene synthase producing linalool and nerol from GPP/NPP substrates. In contrast, its close paralog GmTPS15 exhibited no detectable catalytic activity in either *in vitro* or heterologous in planta assays. Although GmTPS15 produced no detectable volatile products in either *in vitro* assays or transient expression assays in *N. benthamiana* under the tested conditions, we cannot exclude the possibility that it retains activity with alternative substrates, under different assay conditions, or in native soybean cellular contexts where specific cofactors, interacting proteins, or post-translational modifications may be required for proper folding or function. Together, these findings highlight contrasting evolutionary and functional trajectories of two closely related TPS paralogs in soybean.

Across 27 soybean genomes, we resolved a TPS repertoire of 26 loci partitioned into core, near-core, variable, and private classes, with several members exhibiting cultivar-specific losses, suggesting lineage-specific retention and loss within the family. The broad conservation of a 15-gene core set suggests strong evolutionary constraint across soybean accessions, whereas the uneven retention of variable/private TPSs implies relaxed constraints and ecological specialization among accessions. These inferences are supported by our phylogeny and presence/absence profiling that place soybean TPSs into five clades and highlight high-loss members (e.g., GmTPS24/25/26) alongside cultivar-specific absences (e.g., GmTPS1/6/18). Similar phenomena have been observed in diverse plant species. For example, ZmTPS31 and ZmTPS28 were found to be present in only one (CML333) and two (CML6 and Mo18W) varieties, respectively, suggesting that these two genes may be associated with traits specific to these varieties in the analysis of the pan-genome TPS genes in maize (Sun et al., 2023). Comparable gene deletion patterns have also been documented in the pan-genomes of other plants (Song et al., 2024; Sun et al., 2022; Zhang et al., 2025). In our selection pressure analysis, most *GmTPS* genes exhibited Ka/Ks < 1, indicating that soybean have undergone extensive purifying selection at TPS loci, maintaining functional constraints on TPS-mediated defense. In the study of barley, the Ka/Ks of the bHLH gene family was also generally less than 1 (Tong et al., 2025). This suggests that purifying selection is a common evolutionary force acting not only on terpene biosynthesis genes but also on other stress- or disease-resistance-related gene families.

These findings underscore the importance of TPS genes, which is consistent with the well-established roles of terpenoid compounds in plant resistance to environmental stresses (Darragh et al., 2025; Jan et al., 2021; Jing et al., 2021; Shimoda et al., 2012; Wang et al., 2025). The functions of several core *TPS* genes have been elucidated, including *GmTPS5* and *GmTPS18*, which encode geraniol and nerol synthases, respectively, and confer resistance to cotton leafworms (*Spodoptera litura*) in transgenic tobacco plants, as well as *GmTPS21* (*GmAFS*), an α-farnesene synthase involved in defense responses against nematodes and insect pests (Lin et al., 2017; Liu et al., 2014; Zhang et al., 2013). This study selected *GmTPS20*, which was most strongly induced by MeJA and aphid feeding, and its highly homologous *GmTPS15* gene for more in-depth investigation. Subcellular localization experiments suggested that GmTPS20 resides in chloroplast, consistent with the biosynthetic logic of terpene metabolism, as monoterpene biosynthesis typically occurs in plastids via the MEP pathway. This compartmentalization mirrors the biosynthetic logic of terpene metabolism: monoterpene biosynthesis typically occurs in plastids via the MEP pathway (Conart et al., 2023; Zhou and Pichersky, 2020b). Biochemical assays confirmed that GmTPS20 catalyzes linalool formation *in vitro*, and transient expression assays further supported linalool production in planta (Huang et al., 2018; van Schie et al., 2007; Yang et al., 2020). By contrast, GmTPS15 showed no detectable catalytic activity under the tested conditions. Linalool is involved in plant defense against herbivores by attracting their predators or by directly repelling herbivores. For example, the transgenic *Nicotiana tabacum* L. plants overexpressing the cotton GhTPS12 gene, which produced a significantly higher level of (3S)-linalool, showed direct defense responses against *Helicoverpa armigera* female adults and *Myzus persicae* (Huang et al., 2018). Moreover, linalool is a key component with defensive effects against herbivores in many plants. As a volatile cue, linalool can attract parasitic wasps and also holds potential in manipulating plant defense, particularly in the biological control of herbivores through insect-resistant cultivar breeding (He et al., 2022; Shu et al., 2021; Yang et al., 2025a).

A key question that follows is why the closely related paralogs GmTPS15 and GmTPS20 exhibit markedly different capacities for linalool biosynthesis. Previous studies have shown that even a single amino acid mutation can alter the specificity and diversity of the products of monoterpenoid synthases. For instance, in a monoterpene synthase, the stability of the intermediate terpinyl cation and the pinene cation is primarily facilitated by residues A320, N340, and I448. Changes in these residues, whether direct or indirect, will affect the synthesis of monoterpenoids or their mixtures (Lu et al., 2024). Molecular docking analysis provided preliminary insights into the mechanistic basis underlying the divergent catalytic activities of GmTPS20 and GmTPS15. Although the two proteins differ at only a single residue within the immediate GPP-binding region, GmTPS20 is predicted to form a more constrained and polar substrate-binding environment than GmTPS15, potentially enabling tighter substrate anchoring and more defined substrate orientation. In contrast, GmTPS15 appears to accommodate GPP within a comparatively relaxed binding pocket. Together with the low expression of GmTPS15 across most tissues and induction conditions, these features suggest functional divergence following gene duplication, although the evolutionary forces maintaining this divergence require further investigation. Nevertheless, these hypotheses remain speculative and will require validation through site-directed mutagenesis and enzymatic assays.

Given that the closely related paralogs *GmTPS15* and *GmTPS20* display strikingly divergent catalytic activities and expression patterns, similar functional divergence may also occur among other *TPS* genes across soybean accessions. Consistent with this possibility, we have observed comparable patterns for *GmTPS8* and *GmTPS23* (unpublished data). Additional examples further illustrate this diversity: TPS21 has been functionally characterized with a defined enzymatic role, whereas TPS26 represents a private gene present only in the L05 lineage (**Figure 1B**). Notably, even within the same evolutionary clade, *GmTPS5* and *GmTPS18* both retain catalytic activity but produce distinct terpene products. Collectively, these observations suggest that during soybean TPS family evolution, closely related paralogs tend to be differentially retained or functionally diversified, either through selective preservation of one or a few highly similar copies or through alterations at key residues that shift enzymatic activity.

While this study provides a comprehensive identification and initial characterization of the soybean TPS gene family, several limitations remain that define important avenues for future research. Functional analyses were performed for only a subset of TPS members, which limits the extent to which conclusions can be generalized across the entire family; expanding biochemical and expression analyses to the remaining TPS genes will be essential to reveal broader functional trends. Although GmTPS20 was induced by aphid infestation and shown to produce defense-associated volatiles, its ecological role was inferred indirectly, underscoring the need for in planta aphid feeding or performance assays to directly link terpene production with defensive outcomes. In parallel, structural modeling and molecular docking analyses were used to propose candidate residues and binding modes, but these predictions remain hypothetical and require experimental validation, for example through site-directed mutagenesis coupled with enzymatic assays. In addition, TPS activities were assessed in heterologous expression systems, which may not fully recapitulate native regulatory and metabolic contexts in soybean, highlighting the value of complementary analyses in soybean tissues or stable transgenic backgrounds.

## 5 Conclusion

In this study, we resolved the organization and evolutionary constraints of the soybean TPS family and identify mechanistic differences between two closely related paralogs, *GmTPS20* and *GmTPS15*. Presence-absence profiling across 27 genomes partitioned TPS loci into core, near-core, variable, and private classes, with a 15-gene core set suggesting strongevolutionary constraints on conserved TPS functions. We demonstrate for the first time that *GmTPS20* catalyzes linalool formation in soybean, whereas its close homolog *GmTPS15* shows no detectable activity under the tested conditions. Structural modeling and docking analyses further suggest that differences in active-site architecture and pocket-shaping residues may contribute to their divergent catalytic behaviors. These findings provide a framework for functional validation of TPS diversification and offer candidate molecular targets for allele-aware engineering of terpenoid pathways in soybean improvement.

## Data Availability

The original contributions presented in the study are included in the article/**Supplementary Material**. Further inquiries can be directed to the corresponding author.
