# Supplementary figures and images for: Pan-genome analysis of soybean terpene synthase identifies GmTPS20 as a defense-related linalool synthase
Source: Front Plant Sci. 2026 May 15;17:1845603. doi: 10.3389/fpls.2026.1845603 (PMC13220700; doi:10.3389/fpls.2026.1845603)

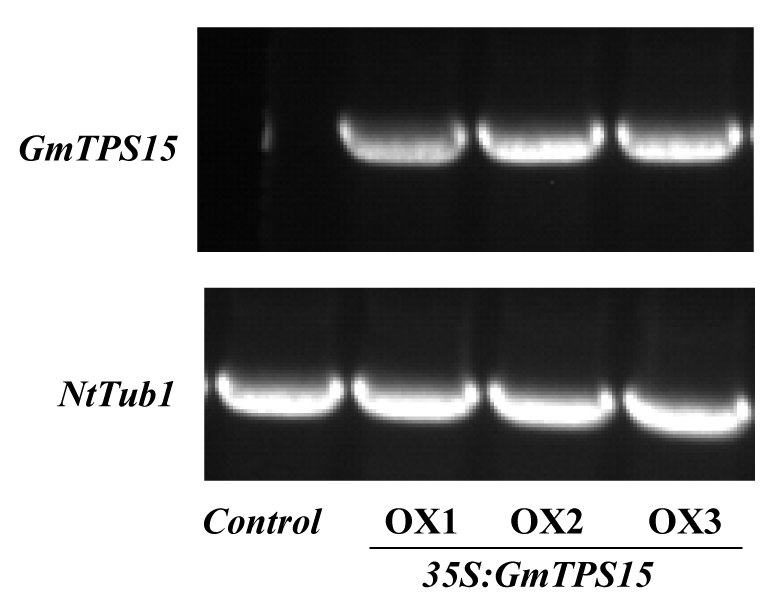

Supplement: Supplementary file 5 [file Image5.tif]

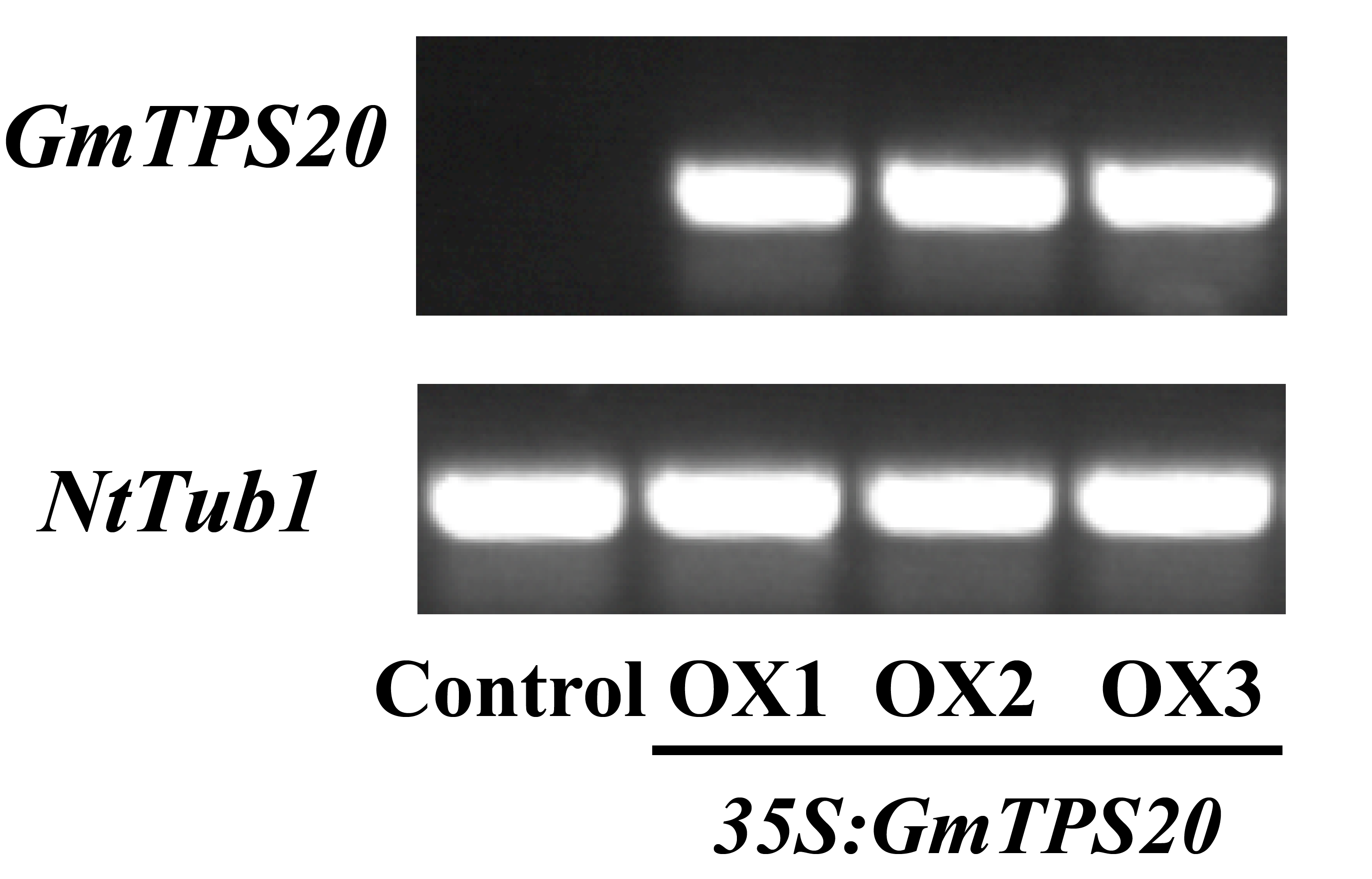

Supplement: Supplementary file 6 [file Image6.tif]

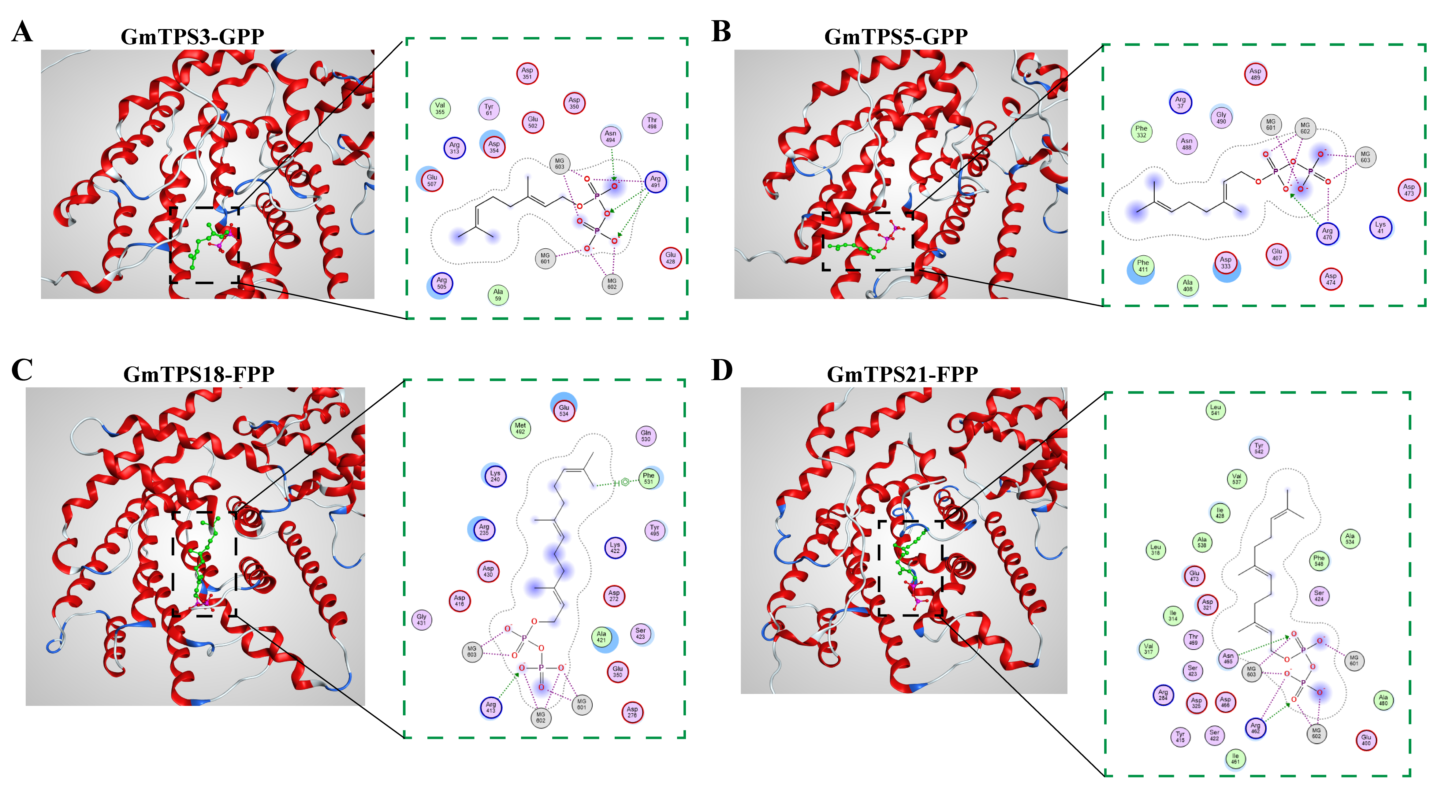

Supplement: Supplementary file 7 [file Image7.tif]
